# Next-Generation Vaccines Leveraging T Cell-Centric Design, Mucosal Immunity, and Trained Innate Immunity for Respiratory and Enteric Pathogens

**DOI:** 10.3390/vaccines14050462

**Published:** 2026-05-21

**Authors:** Md. Abdus Salam, Md. Yusuf Al-Amin, Kasireddy Sudarshan, Aidan Lynch, Victor Reyes, Madeline Stevenson

**Affiliations:** 1 Faculty of Biotechnology & Biomedical Engineering, Rajshahi Medical University, Rajshahi 6100, Bangladesh; 2 Department of Chemistry and Institute for Drug Discovery, Purdue University, West Lafayette, IN 47907, USA; amin50@purdue.edu (M.Y.A.-A.); srkasire@purdue.edu (K.S.); 3 Purdue University Interdisciplinary Life Sciences Graduate Program, Purdue University, West Lafayette, IN 47907, USA; 4 Department of Pharmacy Practice, Purdue University College of Pharmacy, Purdue University, West Lafayette, IN 47907, USA; lynch127@purdue.edu; 5 Department of Biological Sciences, Purdue University, West Lafayette, IN 47907, USA; reyes204@purdue.edu; 6 Department of Agricultural and Biological Engineering, Purdue University, West Lafayette, IN 47907, USA; steve340@purdue.edu

**Keywords:** next-generation vaccines, T cell-centric design, mucosal immunity, trained innate immunity, respiratory pathogens, enteric pathogens

## Abstract

Next-generation vaccines are being developed to elicit durable and cross-protective immune responses against diverse pathogens, particularly those targeting the respiratory and enteric systems. By strategically engaging T cell-centric antigen design, mucosal immune engagement, and induction of trained innate immunity, these innovative platforms are expected to reshape the paradigm of immunoprophylaxis and to offer promising avenues for enhanced protection against complex infectious diseases. Conventional antibody-based vaccines, though effective against many infections, often lack the capacity to induce durable or cross-protective immunity at mucosal surfaces. Advances in antigen design, delivery platforms, and adjuvant technologies now facilitate precise activation of tissue-resident memory T cells and enhancement of mucosal secretory IgA responses, thereby achieving sterilizing immunity at barrier surfaces while reinforcing systemic immune protection. Advanced delivery platforms, including lipid nanoparticles, viral vectors, and nano or liposomal carriers, further refine antigen presentation, enhancing stability, targeting, and overall immunogenicity. Concurrently, progress in understanding trained innate immunity highlights opportunities to induce broad, non-antigen-specific protection through epigenetic and metabolic reprogramming of innate cells. The integration of these adaptive and innate mechanisms may enhance early pathogen control, limits transmission, and strengthens defense against variant and antimicrobial-resistant pathogens across diverse populations. However, translating these immunological insights into safe, scalable, and globally accessible vaccines remains a major challenge. This review explores the emerging conceptual framework of next-generation vaccines that demonstrate partial integration of these axes in preclinical models, though human translation and functional synergy require Phase II validation. It highlights progress toward next-generation vaccines leveraging integrated adaptive and innate immune reprogramming for superior protection against respiratory and enteric pathogens.

## 1. Introduction

Next-generation vaccines include mRNA, DNA, and self-amplifying RNA platforms that encode antigens for endogenous expression and allow rapid sequence-based design. These platforms induce strong humoral and cellular immunity through MHC I/II pathways and are highly adaptable to emerging infectious threats. Viral vectors, nanoparticles, and mucosal routes act mainly as delivery systems that enhance antigen presentation, tissue targeting, and local immune priming, setting them apart from nucleic acid vaccines and from traditional whole-pathogen or protein-subunit approaches [1]. Vaccines that harness T cell-mediated responses, mucosal immunity, and trained innate immunity (TRIM) represent an important shift in preventing respiratory and enteric infections. This strategy may support broader, more durable protection and better limit infection and transmission at mucosal entry sites [2,3]. They shift the focus from preventing severe disease alone to achieving broader, longer-lasting, and more adaptable protection against infection. Next-generation vaccines use advanced technologies and delivery systems to better engage the immune system, aiming to induce strong CD4+ and CD8+ T cell responses, enhance mucosal immunity at the site of infection, and train innate immune cells for faster future responses [4,5]. Powered by technologies such as mRNA, viral vectors, protein–nanoparticle constructs, and novel adjuvants, next-generation vaccines can be rapidly redesigned and targeted to specific tissues [6,7]. This adaptability is especially important for pathogens that mutate rapidly, infect the respiratory or gastrointestinal tracts, or require strong cellular immunity for control, such as influenza viruses, coronaviruses, and enteric pathogens. Intranasal and oral vaccines, together with thermostable formulations and improved delivery systems, may also make immunization more scalable and accessible, particularly in resource-limited settings [8,9].

A clearer link between mechanistic immunology and next-generation vaccine design can be made by moving from immune mapping to rational clinical development. Insights into T-cell differentiation, mucosal tissue-resident memory T cells (TRM) and IgA, antigen-presenting cell programming, B-cell fate, and trained innate reprogramming help guide the choice of antigen, adjuvant, vector, route, and dose. These insights inform epitope-focused antigens, mRNA- or viral-vector platforms, prime-boost regimens, and trained-immunity-based formulations designed to shape defined immune profiles. Several next-generation vaccine platforms that aim to combine T cell-centric, mucosal, and trained innate responses have shown encouraging preclinical results and early-phase clinical signals. For example, intranasal or inhaled ChAd-based candidates, including Ad5/Ad26 vectors targeting TB antigens such as Rv2299c or SARS-CoV-2 spike, have been reported to induce lung T cells, mucosal IgA, and innate immune changes in selected models. These findings suggest potential functional integration, but the evidence remains preliminary and does not yet establish a unified protective mechanism in humans. More generally, encouraging immunogenicity in preclinical models and early trials has not consistently translated into protection, as shown by the first prophylactic hepatitis C vaccine trial, which elicited HCV-specific T cell responses but failed to prevent chronic infection [10]. Potential correlates of protection may include lung TRM frequency, nasal sIgA, and selected monocyte histone modifications, while established surrogates such as pneumococcal anti-capsular IgG remain useful for some vaccines. Regulatory agencies may accept such markers when they are clearly linked to clinical endpoints, but this requires strong supporting evidence. Even so, post-licensure validation is still needed for novel mucosal outcomes, and any Go/No-Go decisions before Phase III should be based on validated, not merely proposed, biomarkers [11].

Beyond their scientific advances, next-generation vaccines may broaden how vaccine performance is assessed by incorporating T cell, mucosal, and innate immune markers alongside conventional antibody measurements. This approach could support more nuanced evaluations of protection, durability, and population-level impact. In the face of emerging infections and ongoing vaccine-preventable diseases, these platforms may offer a path toward more resilient, equitable, and adaptable immunization strategies [12]. Next-generation vaccines may change how protection against respiratory and enteric infections is understood, shifting attention from seroconversion alone to broader, tissue-focused immunity that may help reduce both disease and transmission. They also encourage consideration of T cell, mucosal, and innate immune responses alongside serum antibodies [2,13]. Although the concept is biologically plausible, the systematic integration of all three pillars remains an emerging paradigm rather than a widespread clinical reality today [14].

## 2. Next-Generation Vaccines for Comprehensive Immune Protection

**Figure 1:**
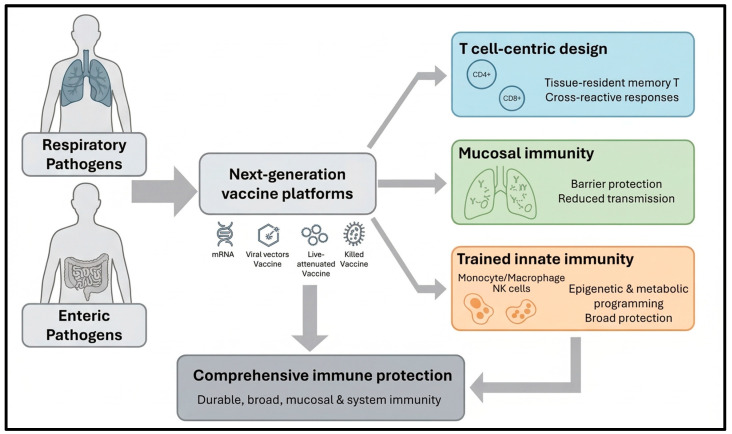
Schematic overview of emerging next-generation vaccine platforms targeting respiratory and enteric pathogens. mRNA, viral-vectored, live-attenuated, and killed vaccines are designed to elicit complementary immune arms: T cell-centric responses to conserved epitopes (including cross-reactive TRMs), mucosal immunity at respiratory and gastrointestinal entry sites to reduce acquisition and transmission and trained innate immunity via epigenetic and metabolic reprogramming of monocytes, macrophages, and NK cells for broad, early, antigen-agnostic protection).

For many years, neutralizing antibody levels in blood have been the main readout of vaccine efficacy. Vaccinology is now expanding beyond this humoral focus toward strategies that engage a broader range of immune responses [15]. Traditional vaccines, such as live attenuated, inactivated, toxoid, and subunit platforms, mainly induce humoral immunity, with some also generating cellular responses. In contrast, next-generation platforms such as mRNA, DNA, viral vectors, and self-amplifying RNA are designed to promote stronger T cell-mediated immunity alongside antibody responses. These platforms also engage innate sensors and support more direct intracellular antigen expression, which can enhance overall immunogenicity [16,17]. Next-generation vaccines may achieve a greater degree of immune integration by coordinating innate and adaptive responses in a more connected manner. They bring together innate pattern recognition and adaptive immunity in ways that may support broader, more durable responses, but the current evidence is still largely preclinical and only partially demonstrates this concept. In particular, claims about direct links between trained innate responses, mucosal TRM maintenance, and IgA class-switching should be stated cautiously, as these relationships still require validation in human studies [18]. The rise of complex pathogens, antigenic variation, and mucosal transmission has exposed the limits of classical vaccine designs and increased interest in responses that provide durable protection and strong immune memory [19]. Respiratory and enteric pathogens such as influenza viruses, SARS-CoV-2, RSV, rotavirus, and norovirus illustrate these limitations, as reinfections and breakthrough disease can still occur despite widespread vaccination and measurable systemic antibody responses [20,21]. Technological advances in structural vaccinology, mRNA and viral-vectored platforms, and systems immunology have improved antigen discovery and delivery, enabling more targeted immune responses. Accordingly, next-generation vaccines should be evaluated not only by traditional serological measures but also by mucosal IgA, tissue-resident T cells, and selected innate immune signatures that may better reflect durable protection (Figure 1) [3,22].

Preclinical studies suggest that some next-generation vaccines can engage T cell-centric, mucosal, and trained innate responses together, but the extent of true functional integration remains variable. For example, rAd-Rv2299c and some MVA/TLR-based approaches have shown encouraging signals in animal models, while early-phase inhaled ChAd COVID-19 studies provide preliminary evidence of multi-layered lung immunity. However, the timing and causal interplay between innate reprogramming and TRM maturation are still not fully defined, especially in humans. Translation remains limited by the need for scalable mucosal delivery, stronger validation beyond BCG-based approaches, and standardized assays of innate immune memory. Longitudinal studies of dendritic cell–T cell interactions and other immune readouts will be important for determining which markers are genuinely useful for clinical development [10].

## 3. T Cell-Centric Strategies for Durable and Resilient Protection

**Figure 2:**
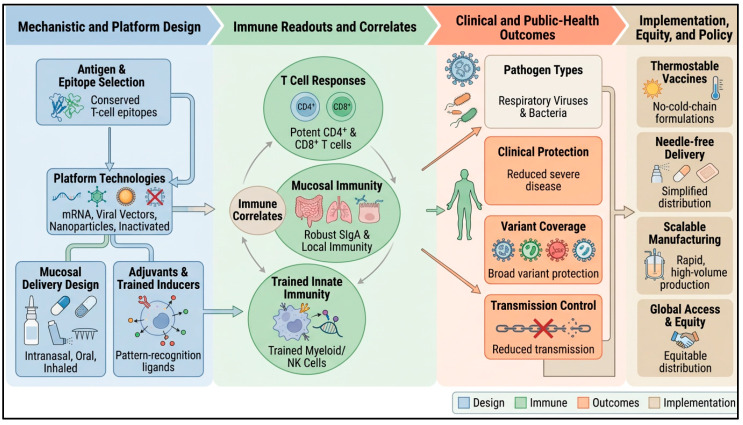
Mechanisms and outcomes of next-generation vaccines integrating T cell, mucosal, and trained innate immunity against respiratory and enteric pathogens. The schematic links mechanistic and platform design/delivery system (antigen/epitope selection, platform technologies such as mRNA, viral vectors, nanoparticles, and live-attenuated/inactivated vaccines, mucosal routes, and adjuvants/trained-immunity inducers), key immune correlates (systemic and tissue-resident T cells, mucosal IgA and local B/T cell responses, and trained innate reprogramming), and clinical/public-health outcomes (reduced severe disease and transmission, broader variant coverage, and improved equity through thermostable, needle-free, scalable vaccines).

T cell-centric vaccines are an emerging approach designed to induce durable, broadly cross-reactive T cell memory, especially CD8+ T cells and tissue-resident memory cells. They may provide longer-lasting, variant-resilient protection and complement waning antibody responses by targeting conserved epitopes less affected by antigenic drift [23,24,25,26]. Modern T cell-centric vaccine design remains largely investigational rather than established clinical practice. Its durability is thought to depend on several interconnected elements: careful epitope selection to identify conserved, broadly recognized targets; inclusion of multiple epitopes to broaden coverage and reduce the risk of escape; optimized formulation and adjuvantation to improve T cell quality rather than quantity; and delivery strategies that promote tissue-resident memory at key sites of infection (Figure 2) [27,28,29,30]. A growing body of work highlights the importance of mucosal TRM cells, especially in the respiratory and gastrointestinal tracts, where many infections begin [31,32]. These cells can provide rapid local protection and may help reduce disease severity and transmission. As immune monitoring improves, measures such as T cell breadth, functionality, phenotype, and localization are increasingly being considered alongside antibody responses when evaluating vaccine candidates. Several platforms are being adapted to strengthen cellular immunity, including peptide or epitope-based vaccines, mRNA constructs, viral vectors, and microneedle-delivered T cell vaccines such as CoronaTcP, with the aim of inducing durable tissue-resident memory and broader variant coverage [33,34,35,36,37,38]. Despite these advances, emerging T cell-centric vaccines face several challenges. Construct designs must balance epitope diversity sufficient for global HLA coverage with practical limits on complexity and manufacturability. Standardized assays are still needed to define which T cell parameters, such as phenotype, localization, and persistence, most accurately predict protection. Furthermore, strong T cell activation carries potential risks of immunopathology or autoimmunity, underscoring the importance of rigorous antigen selection and preclinical safety testing. Finally, while “universal” or pan-pathogen vaccines based on conserved T cell epitopes remain an appealing goal, their broad efficacy across genetically diverse human populations has yet to be conclusively demonstrated [25,39,40].

## 4. Mucosal Immunity at the Body’s Gateways

Mucosal immunity has emerged as a central focus of next-generation vaccine development, as many high-impact pathogens initiate infection at respiratory or gastrointestinal surfaces-the body’s primary portals of microbial entry. These mucosal interfaces represent the largest and most continuously exposed immune environments, constantly engaging with inhaled and ingested agents. It is noteworthy that respiratory and gastrointestinal mucosal systems differ significantly in pH, enzymatic activity, microbiota composition, and immune architecture, with significant implications for the design of mucosal vaccines. The gut lumen is highly acidic, rich in proteolytic enzymes, and densely colonized by a complex microbiota that shapes local IgA-biased, Th2- and Treg-skewed responses in gut-associated lymphoid tissue (GALT). In contrast, the respiratory tract operates at a more neutral pH, with lower enzymatic load and a distinct, sparser microbiota that favors Th1/Th17-biased and dendritic cell-driven responses in bronchial- and nasopharyngeal-associated lymphoid tissues (BALT/NALT). These differences mean that a vaccine formulation or adjuvant optimized for one compartment may not translate directly to the other, even though both tissues share the broader “mucosal immune system” concept [41].

Despite their differences, both respiratory and enteric mucosa share a layered defense system that includes physical barriers, innate effectors, and adaptive components such as secretory IgA, tissue-resident memory T cells, and mucosa-associated lymphoid tissue. This organization supports early pathogen control and helps limit spread beyond the mucosal surface [8,42]. SIgA is the key antibody in mucosal immunity, where it helps block pathogen attachment, neutralize toxins and viruses, and retain immune complexes in mucus. Its polymeric structure and resistance to degradation make it especially effective at epithelial surfaces [43]. Mucosal T cell populations, especially Th1/Th17 subsets and CD8+ tissue-resident memory cells, contribute to rapid recall and local effector responses after re-exposure [44]. Innate mediators such as mucins, defensins, and type I/III interferons help shape the early mucosal environment and support the transition from innate recognition to adaptive effector responses [45,46]. Collectively, these components can limit local replication and transmission, thereby reducing disease severity. Achieving sterilizing or near-sterilizing immunity at mucosal surfaces would be especially valuable for pathogens that spread easily in households, schools, and other close-contact settings [3,9]. This is particularly relevant for respiratory and enteric viruses, where systemic antibody responses alone may not fully prevent reinfection or breakthrough disease. For example, live attenuated influenza vaccines (LAIV) (e.g., FluMist) can induce mucosal IgA and tissue-resident T cells, but their effectiveness against infection can vary across seasons because of antigenic mismatch and prior immunity [47]. Similarly, oral rotavirus vaccines (RV1, RotaTeq) provide partial infection protection (~50–70% in high-income settings) yet excel (>85–95%) against severe disease via heterotypic immunity, underscoring the distinction between sterilizing and disease-mitigating effects [48]. The COVID-19 experience showed that intramuscular vaccines were highly effective at preventing severe disease but were less effective at blocking upper airway infection. This highlights the need for local mucosal responses in addition to systemic immunity if true infection-blocking protection is the goal [11,49,50,51,52]. During Omicron waves, mRNA vaccines (BNT162b2, mRNA-1273) sustained 70–90% protection against hospitalization vs. 40–60% for protein vaccines (Novavax), attributable to robust polyfunctional CD4+/CD8+ T cell immunity targeting conserved epitopes [53]. Vector-based vaccines (Ad26.COV2.S) similarly elicited tissue-resident T cells correlating with milder outcomes despite antibody waning [54]. Considering the distinct features of respiratory and gastrointestinal mucosa, next-generation vaccines are being developed for intranasal, oral, sublingual, or inhaled delivery to target mucosal lymphoid tissues and promote local immune responses. These platforms may help induce tissue-resident memory and mucosal IgA at relevant entry sites (Figure 2). Mucosal priming may also broaden protection across different mucosal surfaces, which is particularly relevant for enteric pathogens such as cholera, rotavirus, and norovirus [3,9,13,55]. The design of these vaccines should reflect the distinct features of each mucosal site, including epithelial barrier integrity, mucus composition, microbiota, and enzymatic activity. Delivery approaches such as nanoparticles, lipid-conjugated antigens, mucoadhesive matrices, and targeted adjuvants are being developed to improve antigen uptake, extend mucosal retention, and favor durable SIgA and tissue-resident T cell responses [9,13,56,57].

## 5. Trained Innate Immunity as a Rapid Frontline Defense

Trained innate immunity (TRIM) is a form of memory-like reprogramming in innate immune cells that can lead to faster and stronger responses on re-exposure, including to unrelated pathogens. Although still incompletely understood, it is best viewed as a supportive mechanism in next-generation vaccine design rather than a primary standalone strategy. Because it may also increase the risk of inflammation, immune dysregulation, or autoimmunity, careful attention to dose, timing, and patient selection remains important [58]. Unlike classical adaptive memory, trained immunity arises in myeloid cells (monocytes, macrophages, dendritic cells), NK cells, and their bone marrow progenitors following exposure to specific microbes, vaccines, or pattern-recognition receptor (PRR) ligands. This stimulus induces lasting epigenetic and metabolic changes, such as histone modifications (e.g., H3K4 trimethylation, H3K27 acetylation) and shifts in cellular metabolism, that prime cells for stronger responses upon re-exposure. Trained innate cells can show higher cytokine production (TNF α, IL 6, IL 1β), better pathogen killing, and improved antigen presentation, with effects that may persist for months through reprogramming of hematopoietic stem and progenitor cells [59,60]. Classic examples include Bacillus Calmette–Guérin (BCG) and other live or inactivated vaccines that confer nonspecific protection against heterologous infections, now attributed largely to trained immunity [61]. More recently, viral vectored and mRNA COVID-19 vaccines have been shown to produce long-term transcriptional and functional reprogramming of myeloid cells, suggesting that innate training contributes to their durability and breadth of protection [62]. These findings support the concept of TRIM-based vaccines (TIbVs), which aim to promote beneficial innate reprogramming alongside conventional adaptive immunity. Such platforms may incorporate PRR ligands, including β-glucans (Dectin 1 agonists), muramyl dipeptide (NOD2 agonist), muramyl dipeptide, or TLR7/8 agonists, into their adjuvant or delivery systems to favor useful epigenetic and metabolic changes while minimizing the risk of inflammation, exhaustion, or other adverse effects. Trained innate immunity as a vaccine strategy has important limitations. A key concern is the possibility of excessive inflammation and immune dysregulation, because TRIM-primed myeloid cells may produce stronger IL-1β, IL-6, and TNF-α responses after restimulation, with potential for tissue damage in chronic or repeated exposure settings. In some contexts, such as chronic infection, autoimmunity, or allergy, this hyperresponsive state may also favor pathogenic Th1/Th17 polarization. Another limitation is durability: TRIM effects are often shorter and less predictable than classical adaptive memory, sometimes lasting only months to about a year after stimuli such as BCG. TRIM is also non-antigen-specific, so its effects are broad but less precisely targeted and may influence responses to commensals, self-antigens, or allergens. In addition, important gaps remain in understanding its signaling pathways, epigenetic and metabolic basis in humans, and interaction with adaptive immunity. The absence of validated biomarkers further complicates dose selection, scheduling, risk stratification, and personalized design [63]. Rational design should take into account the target cell populations, route of administration (systemic vs. mucosal), and booster timing to improve durability and safety (Figure 2) [62,64]. Strategically trained immunity may help narrow the window of vulnerability after exposure, limit early pathogen replication, and complement antigen-specific antibody and T cell responses. It may be especially useful for emerging infections, immune-evasive variants, and high-risk groups such as infants, older adults, and immunocompromised individuals [64]. Deliberate integration of trained immunity principles into vaccine design may involve adjuvants and delivery strategies that promote beneficial innate reprogramming while minimizing excessive inflammation or autoimmunity [65,66]. Achieving this safely requires careful control of dose-response dynamics, the duration of training, and the risk of excessive inflammation or autoimmunity. Key research priorities include defining correlates and biomarkers of trained protection, such as epigenetic signatures, cytokine profiles, and metabolic markers, to support clinical translation of TIbVs [63,67].

Figure 2 illustrates the translational pathway for next-generation vaccines, linking antigen and platform design, mucosal delivery, and adjuvant selection with immune readouts and clinical outcomes. It also highlights the potential relevance of systemic and mucosal T and B cell responses, along with trained immunity, for respiratory and enteric vaccines. Table 1 summarizes the main features of these approaches, including their immune focus, mechanisms, delivery platforms, and representative candidates.

## 6. Functional Integration of T-Cell-Centric, Mucosal, and Trained-Innate Immunity

Conceptual frameworks for next-generation vaccines integrating T cell-centric, mucosal, and trained innate immunity are based mainly on mechanistic plausibility, including mucosal delivery, RNA- or PAMP-based adjuvanticity, and inferred tissue-resident memory or heterologous protection. By contrast, experimentally supported systems demonstrate antigen-specific mucosal TRM and IgA, together with trained–innate signatures and measurable protection in challenge models. The key distinction is whether these three axes are directly measured and causally linked in the same experimental setting rather than inferred from separate readouts. A limited but growing number of platforms show partial multi-axis integration, although the strength of validation remains uneven [72]. Table 2 presents next-generation vaccine platforms with direct or partial experimental evidence of integrated T cell-centric, mucosal, and trained–innate immune responses in preclinical mouse models.

## 7. Platform Technologies for Next-Generation Vaccines

Next-generation vaccine platforms, such as mRNA, DNA, viral vectors, protein nanoparticles, and live-attenuated or inactivated formats, represent the core technology used to encode or present antigens and shape the immune response. In contrast, delivery systems, including lipid nanoparticles, microneedle patches, oral, intranasal, or inhaled formulations, liposomes, virosomes, and mucoadhesive nanoparticles, serve as vehicles or routes that deliver these platforms to the appropriate cells and tissues without changing their fundamental encoding logic. These technologies are increasingly being engineered to combine T cell-centric antigen design with mucosal delivery and trained innate immunity, especially for respiratory and enteric pathogens. This convergence shifts vaccine development beyond purely systemic, antibody-focused approaches toward adaptable platforms that can block infection at entry sites and promote durable, cross-strain cellular and innate protection [4,71]. Modern platforms such as mRNA, self-amplifying RNA, and viral vectors enable rational selection of conserved epitopes and multi-epitope inserts to induce robust CD4+ and CD8+ T cell responses, including tissue-resident memory T cells in the respiratory and gut mucosa [72]. Artificial intelligence-guided antigen design and high-throughput immunoinformatics can prioritize broadly conserved T cell targets, improve HLA coverage, and balance class I and class II presentation to support cross-variant protection against rapidly evolving respiratory viruses and enteric pathogens. In parallel, layer-by-layer and virus-mimicking nanoparticle platforms can co-display multiple antigens and adjuvants in defined geometries, enhancing T cell priming and expanding functional breadth [73]. For respiratory and enteric pathogens, next-generation vaccines increasingly use intranasal, inhaled, oral, or sublingual delivery systems, including polymeric and lipid-based nanoparticles, virus-like particles, and nanotube carriers engineered for mucoadhesion, epithelial transport, and uptake by antigen-sampling cells. These approaches target inductive sites such as NALT and GALT, promoting strong local IgA and tissue-resident T cell responses while preserving systemic immunity. Complementary advances, including microneedle patches and thermostable oral formulations, further improve stability, ease of administration, and access in low-resource settings [12,74]. Adjuvant and carrier design now enables the incorporation of ligands that promote trained innate immunity through targeted activation of monocytes, macrophages, dendritic cells, and NK cells via defined PRR agonists and metabolic-epigenetic reprogramming. Nanoparticle- and scaffold-based delivery systems can co-deliver antigens and innate training cues, eliciting rapid nonspecific defenses that reduce pathogen load and enhance adaptive responses. These modular adjuvant systems allow innate activation to be tailored to different respiratory or enteric threats while preserving safety and scalability [75,76]. Viral vectors and recombinant nanoparticle systems can efficiently deliver antigens to dendritic cells, promoting cross-presentation to cytotoxic T cells together with strong antibody responses [77]. Similarly, protein and peptide vaccines displayed on nanoparticles or virus-like particles, when combined with mucosal adjuvants and delivered orally or intranasally, can induce balanced mucosal and systemic immunity. Advances in thermostable formulations, self-amplifying RNA, and tailored adjuvants allow more precise modulation of innate sensing pathways, supporting durable and well-balanced adaptive responses. Because these platforms are modular, they can be adapted rapidly across pathogens while preserving safety, manufacturability, and consistency in dosing and administration [33,54]. Together, these advances align antibody, T cell, mucosal, and innate responses within a unified design framework, creating a foundation for rapidly adaptable, globally deployable next-generation vaccines. Novel delivery technologies, including microneedle patches, thermostable formulations, and controlled-release systems, further improve feasibility in routine practice and resource-limited settings [78,79]. Table 3 summarizes the distinctions between vaccine platforms and delivery systems in next-generation vaccines.

## 8. Implications of Next-Generation Respiratory Vaccines

The clinical and public health implications of next-generation vaccines for respiratory pathogens are substantial, as these platforms may improve individual protection while also reshaping population-level disease control. By more effectively mobilizing T cell responses, inducing mucosal immunity, and engaging trained innate defenses, such vaccines are expected to reduce severe outcomes, including hospitalization and death, as well as symptomatic and asymptomatic infection. This is especially relevant for influenza, SARS-CoV-2, and RSV, where breakthrough disease remains common despite current vaccines [5,80]. By targeting conserved epitopes and inducing tissue-resident memory at the respiratory mucosa, next-generation platforms may provide broader cross-variant protection and longer-lasting immunity. This could reduce the need for frequent booster campaigns and lessen the clinical burden associated with seasonal surges and variant waves [81]. On the public health level, better control of upper-airway infection and viral shedding could reduce transmission, improving outbreak mitigation in high-risk settings such as long-term care facilities, schools, and healthcare environments. It could also lessen reliance on disruptive nonpharmaceutical interventions during respiratory virus seasons [82]. In addition, intranasal, inhaled, and thermostable formulations have important implications for vaccine equity and global health, as they can simplify logistics, improve acceptability, and expand coverage in resource-limited or remote settings where cold-chain infrastructure and trained personnel are limited [83]. Regulatory and policy frameworks will need to evolve in parallel, incorporating new correlates of protection that capture T cell, mucosal, and innate immune markers rather than relying mainly on serum antibody titers. Trial endpoints should also expand to include reductions in transmission, reinfection, and long COVID-like sequelae, alongside traditional severe disease outcomes. Together, these developments position next-generation respiratory vaccines not only as tools for preventing severe illness, but also for reshaping clinical practice, vaccination schedules, and public health strategies toward more comprehensive and resilient control of respiratory pathogens across diverse populations [84,85].

## 9. Opportunities and Challenges in Next-Generation Enteric Vaccines

Next-generation enteric vaccines offer an important opportunity to reduce disease caused by intestinal pathogens such as *Vibrio cholerae*, *Salmonella*, *Shigella*, *rotavirus*, and enterotoxigenic *Escherichia coli* (ETEC), which continue to cause substantial morbidity and mortality, especially in children in LMICs. These vaccines must also address enteric-specific barriers, including maternal antibody interference, coinfections, malnutrition, and environmental enteric dysfunction, all of which can weaken vaccine responsiveness [86,87]. Next-generation approaches may help overcome these barriers by using oral or rectal mucosal vaccines to induce robust IgA and tissue-resident T cell responses, adjuvant systems tailored to the gut immune milieu, and platforms that incorporate trained immunity to strengthen early-life protection [3,8]. There is also growing interest in microbiome-informed vaccine design, given evidence that gut microbial composition influences vaccine responsiveness [87]. The emergence of novel platforms, including live-attenuated vectors, outer membrane vesicle-based vaccines, and mRNA or nanoparticle delivery systems, offers promising opportunities to overcome the limitations of traditional oral vaccines, particularly poor immunogenicity in individuals exposed to high enteric pathogen loads or malnutrition [88]. Moreover, integrating adjuvants that enhance mucosal immunity, along with multivalent formulations targeting multiple pathogens in a single dose, offers transformative potential for more efficient disease control [89]. However, significant challenges remain in the development of enteric vaccines. Biological barriers, including the harsh gastrointestinal environment, the complex gut microbiome, and the difficulty of inducing durable mucosal immunity across diverse populations, continue to limit consistent vaccine efficacy [90]. Manufacturing scalability, cold-chain dependence, and varying regulatory frameworks across endemic regions further complicate widespread implementation. In addition, the absence of validated correlates of protection for many enteric diseases limits rational vaccine design and hampers rigorous efficacy assessment [91]. Bridging immunological insights with translational research, together with strong public health infrastructure and equitable access strategies, will be essential to realizing the potential of next-generation enteric vaccines [92,93,94].

## 10. Conclusions

Next-generation vaccines that integrate T cell-centric design, mucosal targeting, and trained innate immunity represent a promising but still emerging paradigm in infectious disease prevention. Preclinical studies suggest that these platforms can induce tissue-resident T cells, local IgA, and durable innate reprogramming, but evidence remains limited to animal models and has not yet been confirmed in human trials. Major barriers to clinical translation remain, including the need for standardized correlates of protection, clearer safety benchmarks, and stronger evidence on durability, reactogenicity, and off-target inflammatory effects across diverse populations. Progress is further constrained by route-specific challenges, manufacturing limitations, regulatory uncertainty, and the lack of scalable, thermostable, globally accessible formulations. Overcoming these multifaceted obstacles will be essential before these platforms can be translated into broad clinical and public health use and realize their potential to improve disease control, transmission reduction, and preparedness for future outbreaks.

## Figures and Tables

**Table 1:** Salient features of next-generation respiratory and enteric vaccines.

Immune Focus	Key Mechanisms	Platform Technologies	Candidate Vaccines	Reference
**T cell-mediated responses**	Induce strong, Th1-biased, polyfunctional CD4^+^ and CD8^+^ T cells against conserved epitopes for cytotoxic clearance and durable memory.	Viral vectors (e.g., adenoviral, poxviral) deliver antigen genes to APCs; nucleic acid vaccines (mRNA/DNA) use lipid-based nanoparticles to enhance cellular uptake and antigen expression.	Human-Adenoviral TB or RSV vaccines (preclinical or early clinical stage), mRNA-based RSV or CMV (phase 1–2 trials).	[68,69]
**Mucosal immunity**	Induce local sIgA and tissue-resident T/B cells, engaging mucosal dendritic cells and M cells to strengthen barrier immunity and limit pathogen transmission at respiratory or gut entry sites.	Needle-free oral/intranasal delivery of live-attenuated or inactivated vaccines with particulate or nanocarrier systems (e.g., liposomes, virosomes, mucoadhesive nanoparticles) protects antigen and enhances mucosal retention and uptake.	Oral and intranasal viral-vector or live-attenuated candidates for SARS-CoV-2 and other respiratory pathogens (e.g., NIAID-funded MPV/S-2P, Vaxart-type platforms, in phase 1–2 trials).	[70]
**Trained innate immunity**	Through epigenetic and metabolic changes to enhance cytokine output and broad reactivity, creating a pool of broadly protective innate cells that complement adaptive immunity.	Live-attenuated mycobacterial or viral and engineered BCG-like vaccines activate PRRs and inflammasomes to train innate immunity and deliver antigens for combined trained–innate and adaptive responses.	MTBVAC (live-attenuated *M. tuberculosis*), designed as a next-generation TB vaccine, induces trained immunity and cross-protection in preclinical models and has advanced to early-phase human trials.	[71]

**Table 2:** Direct and partial experimental evidence of integrated T cell-centric, mucosal, and trained innate immunity in preclinical mouse models.

Platform	T-Cell-Centric Axis (Key Evidence)	Mucosal Axis (Key Evidence)	Trained–Innate Axis (Key Evidence)	Reference
Intranasal ChAdOx1 nCoV-19 (SARS-CoV-2 booster post-mRNA prime, mice)—direct evidence in mouse model.	Lung/nasal TRM CD4+/CD8+ T cells (CD103+/CD69+ up to 21.5%); IFN-γ/TNF-α polyfunctional responses	Spike-specific IgA in NALT/BALF; resident B cells/IgA+ plasma cells in URT/LRT	Macrophage/DC influx (CD11b+); myeloid activation in NALT/nasal turbinates post-vaccination	[72]
rBCG-LTAK63 (recombinant BCG, TB mice)—evidence is limited and indirect in preclinical mouse model.	Enhanced CD4+ Th1/Th17 polarization via BMDM co-culture; protection against Mtb	Mucosal delivery boosts lung-resident responses	Inflammasome activation in BMDMs; broad innate reprogramming enhances T cell priming	[73]
rBCG-PPE27 (recombinant BCG, COVID combo, mice)—evidence is limited and indirect in preclinical mouse model.	Boosted Th1 cytokine by vaccine-specific T cells; heterosubtypic protection	Mucosal IgG/IgA acceleration with COVID vaccine	Innate activation augments heterologous pathogen responses	[74]

**Table 3:** Distinctions between vaccine platforms and delivery systems for next-generation vaccines.

Aspect	Platforms	Delivery Systems
Definition	Core immunogenic construct: the antigen–adjuvant–immunomodulatory unit.	Vehicle and route that determine site of administration.
Key examples	- mRNA-LNP platforms (self-adjuvanting antigen-encoding RNA) - Viral-vector platforms (e.g., adenovirus) - Protein-subunit + adjuvant platforms (e.g., structure-guided antigens with TLR ligands) - Whole-cell or live-attenuated platforms (e.g., BCG, MTBVAC) - Trained-immunity-based platforms (TIbVs: polybacterial, β-glucan-rich, PAMP-enriched formulations).	- Parenteral routes: intramuscular, subcutaneous, intradermal - Mucosal routes: intranasal, inhaled, oral, sublingual/buccal - Device-enabled systems: microneedle patches, jet injectors, aerosol/Opti-Nebulizer devices, oil-in-water or LNP-based intranasal formulations.
Primary function	Determine antigen specificity, T-cell/antibody polarization, and innate-adaptive crosstalk (including trained–innate reprogramming).	Determine local immune milieu (e.g., lung-resident vs. systemic), antigen retention, cellular uptake (e.g., dendritic cells, macrophages), and initial cytokine milieu.
Immune axis impact	- Shapes T-cell lineage (Th1/Th2/Th17/Tissue resident T memory cell) - Determines B-cell isotype and affinity maturation - Can drive trained–innate programming in myeloid cells and progenitors.	- Influences whether responses are systemic (e.g., IM) or mucosal-localized (e.g., intranasal) - Affects homing and tissue residency of T and B cells - Modulates magnitude and kinetics of innate activation.
Clinical relevance	Drives vaccine “type” (e.g., mRNA-based, vector-based, TIbV) and long-term immune profile, including durability and cross-protection.	Drives route-specific safety and efficacy (e.g., needle-free mucosal vs. intramuscular), dose-sparing potential, and user acceptability/adherence.

## Data Availability

No new data were created or analyzed in this study. Data sharing is not applicable to this article.

## Abbreviations

The following abbreviations are used in this manuscript:

TRIM	Trained innate immunity
TRM	Tissue-resident memory T cells
IgA	Immunoglobulin A
SIgA	Secretory immunoglobulin A
Th1	T helper 1 cells
Th17	T helper 17 cells
NK	Natural killer (as in NK cells)
PRR	Pattern-recognition receptor
PAMP	Pathogen-associated molecular pattern
TLR	Toll-like receptor
STING	Stimulator of Interferon Genes
NALT	Nasal-associated lymphoid tissue
GALT	Gut-associated lymphoid tissue
TNF	Tumor necrosis factor
IFN	Interferon
IL	Interleukin (for example, IL-6, IL-1)
BCG	Bacillus Calmette–Guérin
RSV	Respiratory syncytial virus
hMPV	Human metapneumovirus
TIbV	Trained immunity-based vaccine
NOD2	Nucleotide-binding oligomerization domain containing 2
mRNA	Messenger RNA
RIG	Retinoic acid-inducible gene
HLA	Human leukocyte antigen
MHC	Major histocompatibility complex
SARS-CoV-2	Severe acute respiratory syndrome coronavirus 2
Corona TcP	Coronavirus T cell polymer (or peptide/patch)
LAIV	Live attenuated influenza vaccine
LMICs	Low- and middle-income countries
ETEC	Enterotoxigenic Escherichia coli
